# FLT3 Mutations in Early T-Cell Precursor ALL Characterize a Stem Cell Like Leukemia and Imply the Clinical Use of Tyrosine Kinase Inhibitors

**DOI:** 10.1371/journal.pone.0053190

**Published:** 2013-01-24

**Authors:** Martin Neumann, Ebru Coskun, Lars Fransecky, Liliana H. Mochmann, Isabelle Bartram, Nasrin Farhadi Sartangi, Sandra Heesch, Nicola Gökbuget, Stefan Schwartz, Christian Brandts, Cornelia Schlee, Rainer Haas, Ulrich Dührsen, Martin Griesshammer, Hartmut Döhner, Gerhard Ehninger, Thomas Burmeister, Olga Blau, Eckhard Thiel, Dieter Hoelzer, Wolf-Karsten Hofmann, Claudia D. Baldus

**Affiliations:** 1 Charité, University Hospital Berlin, Campus Benjamin Franklin, Department of Hematology and Oncology, Berlin, Germany; 2 Goethe University Hospital, Department of Medicine II, Hematology and Oncology, Frankfurt/Main, Germany; 3 Department of Hematology and Oncology, University of Düsseldorf, Düsseldorf, Germany; 4 Department of Hematology and Oncology, University of Essen, Essen, Germany; 5 Department of Hematology and Oncology, Klinikum Minden, Minden, Germany; 6 Department of Internal Medicine III, University of Ulm, Ulm, Germany; 7 Department of Hematology and Oncology, University of Dresden, Dresden, Germany; 8 University Hospital Mannheim, Department of Hematology and Oncology, Mannheim, Germany; University of North Carolina at Chapel Hill, United States of America

## Abstract

Early T-cell precursor acute lymphoblastic leukemia (ETP-ALL) has been identified as high-risk subgroup of acute T-lymphoblastic leukemia (T-ALL) with a high rate of *FLT3*-mutations in adults. To unravel the underlying pathomechanisms and the clinical course we assessed molecular alterations and clinical characteristics in a large cohort of ETP-ALL (n = 68) in comparison to non-ETP T-ALL adult patients. Interestingly, we found a high rate of *FLT3*-mutations in ETP-ALL samples (n = 24, 35%). Furthermore, *FLT3* mutated ETP-ALL was characterized by a specific immunophenotype (CD2+/CD5-/CD13+/CD33-), a distinct gene expression pattern (aberrant expression of *IGFBP7*, *WT1*, *GATA3*) and mutational status (absence of *NOTCH1* mutations and a low frequency, 21%, of clonal TCR rearrangements). The observed low *GATA3* expression and high *WT1* expression in combination with lack of *NOTCH1* mutations and a low rate of TCR rearrangements point to a leukemic transformation at the pluripotent prothymocyte stage in *FLT3* mutated ETP-ALL. The clinical outcome in ETP-ALL patients was poor, but encouraging in those patients with allogeneic stem cell transplantation (3-year OS: 74%). To further explore the efficacy of targeted therapies, we demonstrate that T-ALL cell lines transfected with FLT3 expression constructs were particularly sensitive to tyrosine kinase inhibitors. In conclusion, *FLT3* mutated ETP-ALL defines a molecular distinct stem cell like leukemic subtype. These data warrant clinical studies with the implementation of *FLT3* inhibitors in addition to early allogeneic stem cell transplantation for this high risk subgroup.

## Introduction

T-cell acute lymphoblastic leukemia (T-ALL) is an aggressive leukemia accounting for 10–15% of childhood and 25% of adult ALL cases. Based on molecular studies, T-ALL can be divided into at least four molecular-cytogenetic subgroups, i.e. the *TAL/LMO*, the *TLX/HOX11*, the *TLX3/HOX11L2* and the *HOXA* subgroups [1]–[3]. Apart from these genetic subgroups, a fifth subgroup of T-ALL cases with developmental arrest at a very early stage of T-cell development was defined by a characteristic early T-cell precursor (ETP) signature in pediatric T-ALL [4]. This T-ALL subtype, termed as ETP-ALL, is described by an immature surface immunophenotype: absence of CD1a and CD8 expression, weak CD5 expression and expression of one or more myeloid-associated and/or stem cell-associated markers. In addition, an increased genomic instability, a high frequency of remission failures and hematologic relapse characterize this highly unfavorable T-ALL subgroup in pediatric patients [4].

Oncogenic alterations that lead to a differentiation arrest at specific stages of T-cell development are well known for specific subgroups of T-ALL. Of these, the overexpression of the orphan homeobox proteins *TLX1* and *TLX3* lead to a maturation block due to ETS1-mediated TLX recruitment to the Eα core [5]. Most recently, for the group of ETP-ALL a mutational spectrum similar to acute myeloid leukemia (AML) was observed, however no single genetic alterations could be tracked down [6]. For the majority of T-ALL, activation of *NOTCH1* signalling is a driving force in the pathogenesis [7]. Activating *NOTCH1* mutations have been found in more than 60% of T-lineage leukemias and result in a ligand-independent release of the intracellular NOTCH1 domain, which subsequently translocates to the nucleus, where it acts as transcriptional co-activator [8]–[11]. Various groups have shown that activated *NOTCH1* signalling causes activation of downstream targets including *HES1, DTX1, PTCRA*, and *MYC* and clinical studies have explored gamma secretase inhibitors (GSI) as targeted therapeutic strategy in T-ALL [12]–[14].

In contrast to the high frequency of *NOTCH1* mutations, activating *FLT3* mutations (*FLT3*mut) occur only in a very low frequency of T-ALL cases (1–3%), but were evaluated in only limited patient series [15]–[17]. In contrast, mutations of the *FLT3* gene, including internal tandem duplications (ITD) and tyrosine kinase domain (TKD) mutations, are one of the most frequent somatic alterations in AML. About one third of AML patients harbor these alterations, which are associated with a poor prognosis in both, adult and pediatric AML [18], [19]. These findings have promoted the use of tyrosine kinase inhibitors (TKI) in patients with *FLT3* mutated AML [20], [21].

Recently, we have characterized ETP-ALL as a subgroup of early T-ALL in adults [22]. To unravel the underlying pathomechanisms of ETP-ALL and to extend our insights on *FLT3mut* ETP-ALL, we performed a comprehensive molecular and clinical study on a large cohort of adult ETP-ALL patients. We were able to demonstrate that *FLT3mut* ETP-ALL could be classified by its specific immunophenotype and distinctive stem cell like characteristics. Moreover, T-lymphoblastic cells transfected with FLT3-ITD constructs were particular sensitive to tyrosine kinase inhibition making this a new and potentially useful therapeutic option.

## Materials and Methods

### Patients and treatment

We screened 1241 peripheral blood and bone marrow samples of T-ALL patients that were sent to the central diagnostic reference laboratory of the German Acute Lymphoblastic Leukemia Multicenter Study Group (GMALL). Most cases were characterized with monoclonal antibodies to precursor cells (CD10, CD34, CD117, TdT and HLA-DR) and with a selection of lymphoid-associated antigens including surface and cytoplasmic (c) antigens (cCD79a, CD22, cIgM, CD19, CD20, CD24, CD3, TCR, CD2, CD5, CD4, CD8, CD7, CD1a) and myeloid-associated antigens including myeloperoxidase (MPO), CD13, CD33, CD65s, CD15, CD14, CD64. An antigen was considered positive, if they were expressed in ≥20% of leukemic cells (10% for cytoplasmic antigens). Classification of ETP-ALL was based on the immunophenotypic diagnostic criteria as originally described [4]: CD5 <75%; CD1a and CD8 <5%; CD117, CD34, HLA-DR, CD13, CD33, and CD65s >25%. CD11b was not determined (Suppl. Table S1). Of all immunophenotypically identified ETP-ALL patients (n = 142), sufficient material for further investigations was available in 68 cases. Sixteen of these 68 patients were already included in a previous work [22]. For 52 of these 68 patients clinical follow-up data were available. The median follow-up was 9.4 months (range: 0–124.6 months). Most patients were treated according to protocols of the GMALL study group (43/46, 93% by medical report, Table 1). In addition, 94 T-ALL patients from the GMALL trial 07/2003 were used as reference group, of which nine patients showed an ETP-ALL immunophenotype and were included in the cohort of 68 ETP-ALL patients [23], [24]. Of the remaining 85 non-ETP T-ALL patients, 17 had an immunophenotype of early T-ALL, 15 of mature T-ALL, and 53 of thymic T-ALL. All patients gave written informed consent to participate in the study according to the Declaration of Helsinki. The studies were approved by the ethics board of the Johann Wolfgang Goethe-Universität Frankfurt/Main, Germany.

**Table 1:** Gene expression levels in ETP-ALL compared to non-ETP T-ALL.

Expression		ETP-ALL (N = 68)	non-ETP T-ALL (N = 85)	P-value
***BAALC***	median (range)	0.69 (0.0–27.1)	0.08 (0.0–160.3)	<.001
***IGFBP7***	median (range)	1.24 (0.01–4.2)	0.49 (0.0–16.2)	.009
***WT1***	median (range)	0.53 (0.0–4.2)	<0.01 (0.0–1.6)	<.001
***ERG***	median (range)	1.16 (0.0–18.6)	10.69 (0.5–136.7)	<.001
***MN1***	median (range)	4.59 (0.0–33.1)	0.66 (0.01–2.7)	<.001
***BCL11B***	median (range)	0.09 (0.0–1.4)	0.44 (0.0–9.9)	<.001
***GATA3***	median (range)	2.11 (0.0–27.3)	3.91 (0.3–32.4)	.005
***MEF2C***	median (range)	0.50 (0.0–5.1)	0.20 (0.0–1.7)	.001

P values were calculated by Mann-Whitney-U-test.

### Nucleic acid preparation and molecular characterization

Pretreatment bone marrow and peripheral blood samples from patients were used for DNA and total RNA extraction using TRIzol (Life Technologies, Grand Island, NY, USA) according to the manufacturer's protocol with minor modifications. Complementary DNA (cDNA) was synthesized using 500 ng of total RNA and avian myeloblastosis virus reverse transcriptase (RT-AMV; Roche, Mannheim, Germany) in the presence of RNase inhibitor (RNasin; Roche, Mannheim, Germany).

The samples were investigated by comparative real-time PCR (RT-PCR) for expression of eight genes (*BAALC*, *ERG*, *IGFBP7*, *WT1*, *MN1, GATA3, BCL11B*, and *MEF2C*). The mRNA expression levels for *WT1* [25], *BAALC* [24], *IGFBP7* [26], and *ERG* [24] were determined by RT-PCR as previously described. *MN1*-primers were designed as reported [27]. Primer sequences for the expression analysis of *GATA3*, *BCL11B* and *MEF2C* are available on request.

The *NOTCH1* mutation status was defined by direct sequencing of the N-terminal and the C-terminal region of the HD domain, the N-terminal and the C-terminal region of the PEST domain, and the TAD domain [28], [29].

*WT1* mutations were analysed as recently reported [25]. *FLT3mut* (ITD/TKD) were analyzed using a commercially available FLT3 mutation assay (InVivoScribe Technologies, San Diego, USA). The TCR rearrangement status was assessed by the IdentiCloneTM TCRG Gene Clonality Assay (InVivoScribe Technologies, San Diego, USA).

### Statistical analysis

Differences in the clinical characteristics were tested by the Pearson χ^2^ test. For overall survival (OS) in the different subgroups, Kaplan-Meier curves were created and compared by the Log-rank test. OS was calculated from the time-point of study entry to the time-point of death or last follow-up (censored).

The statistical difference of gene expression between two independent groups was tested by the nonparametric Mann-Whitney-U-test. Differences in the mutation rates were analyzed by the Pearson χ^2^ or the Fisher's exact test. For all tests a *P*-value<0.05 (two-sided) was considered to indicate a significant difference. All calculations were performed using the SPSS software version 19 (SPSS Inc., Chicago, IL, USA) and GraphPad Prism® software version 5 (GraphPad Software Inc., LA Jolla, CA, USA).

### Cell culture and chemicals

The human mature T-cell leukemia cell lines Jurkat, BE13 and MOLT-4 were obtained from the German Resource Center for Biological Material, DSMZ (Braunschweig, Germany) and previously characterized on a molecular level [30]. They were grown in RPMI media with 10% fetal bovine serum. All cell lines were cultured at 37°C in a 5% CO2 humidified chamber. TKI258 was a kind gift from Novartis (Basel, Switzerland). Tyrosine kinase inhibitors Sorafenib and PKC412 were purchased from Alexis/Enzo Life Sciences (BAY 43-9006; Loerrach, Germany) and LC Laboratories (Woburn, MA, USA) respectively. The chemotherapeutic agent cytarabine (AraC) was provided by Merck Chemicals (Darmstadt, Germany).

### Plasmid constructs and transfection

For transduction of Jurkat, BE13 and MOLT-4, 2×10^6^ cells were transfected with either FLT3-ITD, or FLT3-wt expression constructs and with the empty vector as a control, using the Nucleofector systems (Lonza Cologne AG, Cologne, Germany) according to the manufacturer's recommendations. The final concentration of the constructs and the empty vector control was 2 µg. FLT3-wt and FLT3-ITD expression constructs were previously described [31].

### Cell proliferation assay

Cell proliferation was measured with the WST-1 reagent according to the manufacturer's instructions (Roche Diagnostics GmbH, Germany). Briefly, 48 hours (hrs) after transfection with FLT3 constructs and empty vector control, the cells were seeded in a 96-well plate with 2×10^5^/well. Subsequently, the cells were cultured for 48 hrs with Sorafenib, PKC412, TKI258 and AraC as a chemotherapy agent or Dimethylsulfoxid (DMSO) as negative control. Absorbance was measured after 48 hrs by optical density absorption analyses at 450 nm using an ELISA multiplate reader.

The 50% growth inhibitory concentrations (IC_50_) of Sorafenib, PKC412 and TKI258 were determined by plotting the logarithm of the drug concentrations (Sorafenib: 0–500 µM, PKC412: 0–18 nM, TKI258: 0–500 nM) and the growth rate of the cells treated with FLT3-ITD or FLT3-wt constructs and empty vector, using the WST-1 assay.

### Apoptosis assay

The cellular apoptosis was measured transfected with FLT3-ITD or FLT3-wt constructs and with the empty vector. Briefly, after 48 hrs treatment with Sorafenib, PKC412, TKI258, and AraC cells were labelled with Annexin V and 7-amino-actinomycin D (7-AAD), using Annexin V Apoptosis Detection Kit (BD Pharmingen, Heidelberg, Germany) and then analyzed by FACS Calibur (Becton-Dickinson) to determine the percentage of apoptotic cells.

## Results

### Characteristics and clinical outcome of adult ETP-ALL patients

**Figure 1:**
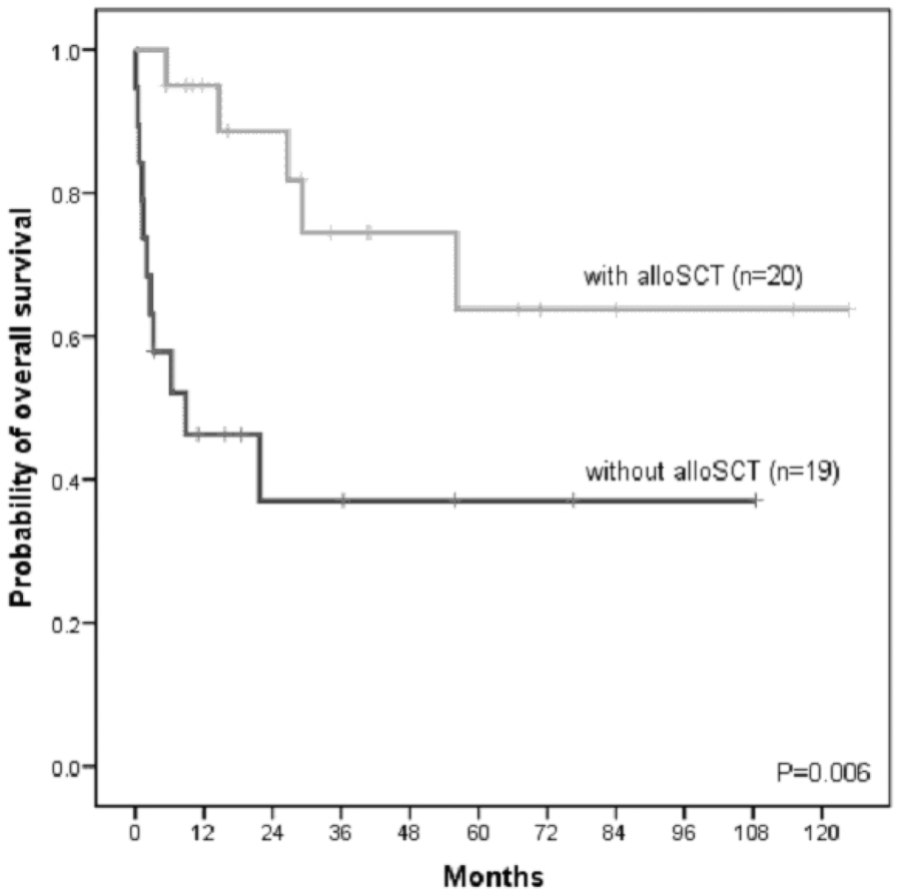
Kaplan Meier analysis of overall survival in adult ETP-ALL patients receiving chemotherapy only (without alloSCT) or undergoing alloSCT. P-value was calculated by the Log-Rank test. Abbreviations: alloSCT, allogeneic stem cell transplantation.

Based on the ETP-ALL specific immunophenotype, we identified pre-treatment samples of 68 newly diagnosed ETP-ALL patients. The median age was 38 years (range: 17–74 years). More patients were male (81%). Of all 68 ETP-ALL patients, follow-up data were available in 52 patients. Forty-five patients were treated according to a GMALL-like protocol, three patients to an AML-like protocol. Fifty-eight percent of patients achieved a complete remission (CR) after induction therapy (Supplementary Table S2). The cumulative 3-year OS was 60%. We further examined outcome with respect to the treatment of chemotherapy only or the allocation to allogenic stem cell transplantation (alloSCT). With the limitation of a potential selection bias for patients undergoing alloSCT, we observed for ETP-ALL patients receiving an alloSCT a favorable outcome (n = 20; 3-year OS: 74%) compared to ETP-ALL patients that were treated with chemotherapy only (n = 19; 3-year OS: 37%, P = 0.006; Figure 1). To address the potential selection bias we have also performed a landmark analysis with a time to transplant of two months. In this analyses patients undergoing alloSCT showed a favourable however not significant benefit compared to patients only receiving chemotherapy.

### Aberrant gene expression and mutational analyses in ETP-ALL compared to non-ETP T-ALL

To further characterize ETP-ALL on the molecular level, we analyzed this large ETP-ALL cohort for the expression of selected genes involved in the pathogenesis and with prognostic implications in adult acute leukemia [22]. *BAALC* and *IGFBP7* were higher expressed in ETP-ALL compared to non-ETP T-ALL patients (*BAALC*, 8.6-fold, P<.001; *IGFBP7*, 2.5-fold, P = .009). Furthermore, expression levels of *WT1* and *MN1* were higher in ETP-ALL compared to non-ETP T-ALL (*WT1*, P<.001; *MN1*, 7-fold, P<.001). Additionally, expression of *MEF2C*, a gene associated with ETP-ALL [32], was significantly higher in ETP-ALL versus non-ETP T-ALL (2.6-fold, P = .001). As critical players in the differentiation program of T-lymphopoiesis, we explored the expression of the transcription factors *GATA3*, required for the development of normal ETPs [33], and *BCL11B*, necessary for the subsequent T-cell lineage commitment [34]. Both, *GATA3* and *BCL11B*, were lower expressed in ETP-ALL compared to non-ETP T-ALL (1.9-fold, P = .005; and 4.9-fold, P<.001; respectively). Similarly, ETS transcription factor *ERG* was also significantly downregulated in ETP-ALL vs. non-ETP T-ALL (9.2-fold, P<.001; Table 1) [35].

The analysis of the TCR rearrangement status revealed that 40 ETP-ALL patients (59%) lacked clonal TCR rearrangements, while 28 patients (41%) showed a monoclonal status. In contrast, 66 (78%) of non-ETP T-ALL patients showed clonal TCR rearrangements, whereas only 18 of these patients (22%) lacked monoclonal TCR rearrangements (Table 2). In addition, differences of the *NOTCH1* and *FLT3* mutation status between ETP-ALL and non-ETP T-ALL cases were explored. We found a low rate of *NOTCH1* mutations in the ETP-ALL subgroup (n = 10/68, 15%), whereas *NOTCH1* mutations were more frequent (40%) in non-ETP T-ALL patients (P<.001). In contrast, we found a high rate of *FLT3* mutations in ETP-ALL compared to non-ETP T-ALL patients: 24 of the 25 *FLT3* mutations were found in the ETP-ALL group, displaying a frequency of 35.3%, whereas non-ETP T-ALL showed a *FLT3* mutations frequency of only 1.2% (P<.001). Ten cases (6.5%) had TKD mutations, of which nine occurred in the ETP-ALL group (P = .003). ITD mutations were found in 15 cases, all belonging to the ETP-ALL group (P<.001, Table 2). In a multivariate analysis, *NOTCH1* mutation status, low expression of *BAALC*, *WT1*, *ERG*, *IGFBP7*, and TCR rearrangement had no additional prognostic impact in the subgroup of ETP-ALL.

**Table 2:** Mutational events in ETP-ALL compared to non-ETP T-ALL.

Mutation Status		ETP-ALL	non-ETP T-ALL	P-value
**TCR rearrangement**	monoclonal	28 (41%)	66 (79%)	<.001
**n = 153**	polyclonal	40 (59%)	18 (21%)	
***NOTCH1***	mut	10 (15%)	35 (41%)	<.001
**n = 151**	wt	56 (85%)	50 (59%)	
***FLT3*** ** total**	mut	24 (35%)	1 (1%)	<.001
**n = 153**	wt	44 (65%)	84 (99%)	
***FLT3*** ** ITD**	mut	15 (22%)	0 (0%)	<.001
	wt	53 (78%)	85 (100%)	
***FLT3*** ** D835**	mut	9 (13%)	1 (1%)	.003
	wt	59 (87%)	84 (99%)	

P values were calculated by Pearson's Chi-square test and Fisher's exact test, respectively.

### Immunophenotype of *FLT3*mut ETP-ALL compared to *FLT3*wt ETP-ALL

In addition to the distinct immunophenotype, *FLT3*mut ETP-ALL showed specific immunophenotypic and molecular characteristics compared to *FLT3*wt ETP-ALL. In this study, 83% (20/24) of *FLT3*mut ETP-ALL patients were positive for CD117, compared to only 28% (13/44) of *FLT3*wt ETP-ALL cases (P<.001). Furthermore, *FLT3*mut ETP-ALL had a higher rate of positivity for CD2 (88% vs. 30%, P<.001) and CD13 (100% vs. 37%, P<.001) compared to *FLT3*wt ETP-ALL patients. *FLT3*wt ETP-ALL was characterized by expression of CD5 (54% vs. 4%, P<.001) and CD33 (54% vs. 4%, P<.001; Figure S1 in Supplementary Figures).

A recent study described the immunophenotype of TdT+/CD7+/CD13+/CD34+/CD117+ as highly specific for the prediction of *FLT3* mutations in an unselected cohort of T-ALL [17]. In our ETP-ALL cohort, 75% (18/24) of patients with *FLT3* mutations showed this immunophenotype, while only 7% (3/44) without *FLT3* mutations displayed this phenotype. Another *FLT3* mutation associated marker profile (sCD3-/CD117+/CD34+/CD62L+/CD56-/CD2+/CD7+/CD1a-/CD4-/CD5-/CD8-/CD13+ [36] can be adopted to 71% (17/24) of our *FLT3*mut ETP-ALL patients; this profile was highly specific for *FLT3* mutations without a false prediction. Here we established the combination of CD2+/CD5-/CD13+/CD33-, able to detect 21 of the 24 *FLT3*mut ETP-ALL patients, as highly sensitive (88%) and specific (95%) algorithm (Table 3).

**Table 3:** Combinations of antigens as a surrogate marker for *FLT3* mutations in ETP-ALL.

		FLT3mut (n = 24)	FLT3wt (n = 44)	P-value	Sensitivity	Specificity
**CD117**	pos	20	13	**<.001**	83%	70%
	neg	4	31			
**TdT+/CD7+/CD13+/CD34+/CD117+** §	pos	18	3	**<.001**	75%	93%
	neg	6	41			
**CD117/CD34+/CD62L+/CD56/CD7+/CD2+/CD5-/CD13+** #	pos	17	0	**<.001**	71%	100%
	neg	7	44			
**CD2+/CD5-/CD13+/CD33-** &	pos	21	2	**<.001**	88%	95%
	neg	3	42			

Abbreviations:

§ combination of markers suggested by Hoehn *et al.* [17],

# combination of markers suggested by Paietta [36],

& combination of markers suggested in this paper. All combinations were adapted to the subgroup of ETP-ALL.

### Molecular characteristics of *FLT3*mut ETP-ALL in contrast to *FLT3*wt ETP-ALL

*FLT3*mut ETP-ALL showed a specific gene expression profile compared to *FLT3*wt ETP-ALL. Higher expression levels of *WT1* (2.2-fold, P = .003) and lower expression of *IGFBP7* (0.11-fold, P<.001) were characteristic for *FLT3*mut ETP-ALL. Remarkably, *FLT3*mut ETP-ALL had a significantly lower expression of the T-cell transcription factor *GATA3* compared to *FLT3*wt ETP-ALL (P<.001). All except one *FLT3*mut ETP-ALL case had *GATA3* expression levels below the median. No significant differences were found for *BAALC*, *ERG*, *MN1*, *BCL11B*, and *MEF2C* (Table 3). As for other lymphoblastic leukemias described [37], [38], *FLT3* itself is overexpressed in ETP-ALL. In this subgroup *FLT3*mut ETP-ALL showed a higher expression compared to *FLT3*wt ETP-ALL samples (P<.01, Figure S2 in Supplementary Figures).

TCR rearrangement analysis demonstrated that *FLT3*mut ETP-ALL patients predominantly lacked clonal TCR rearrangements. Only 21% of the *FLT3*mut ETP-ALL patients in contrast to 52% of the *FLT3*wt ETP-ALL patients showed a TCR rearrangement (P = .01). In addition, none of the *FLT3*mut ETP-ALL patients showed a *NOTCH1* mutation, while 23% (10/44) *FLT3*wt ETP-ALL had *NOTCH1* mutations (Table 4).

**Table 4:** Molecular characteristics of *FLT3*mut ETP-ALL versus *FLT3*wt ETP-ALL patients.

A Expression		FLT3mut (n = 24)	FLT3wt (n = 44)	P-value
***WT1***	median (range)	**0.78** (0.2–4.2)	**0.36** (0.0–3.4)	**.003**
***IGFBP7***	median (range)	**0.30** (0.02–4.0)	**2.52** (0.1–9.9)	**<.001**
***GATA3***	median (range)	**0.06** (0.0–5.7)	**5.82** (0.0–6.7)	**<.001**
*BAALC*	median (range)	0.44 (0.01–17.7)	0.95 (0.0–27.1)	.41
*ERG*	median (range)	0.94 (0.2–18.6)	1.56 (0.0–4.2)	.16
*MN1*	median (range)	6.35 (0.3–16.8)	7.42 (0.0–33.1)	.29
*BCL11B*	median (range)	0.20 (0.0–1.4)	0.05 (0.0–1.0)	.09
*MEF2C*	median (range)	0.71 (0.02–2.2)	0.46 (0.0–5.1)	.22
**B Mutation Status**				
***NOTCH1***	mut	0 (0%)	10 (23%)	**.01**
	wt	24 (100%)	34 (77%)	
**TCR status**	monoclonal	5 (21%)	23 (52%)	**.01**
	polyclonal	19 (79%)	21(48%)	

A: P-values were calculated by Mann-Whitney-U-test.

B: P-values were calculated by Pearson's Chi-square test and Fisher's exact test, respectively.

### Clinical characteristics of *FLT3* mutated ETP-ALL patients

With respect to clinical characteristics, no differences were observed between the *FLT3*mut ETP-ALL and the *FLT3*wt ETP-ALL patients regarding sex and age. The response to induction therapy was similar between both groups (CR: 13/21 vs. 13/24). Three of the 24 *FLT3*mut ETP-ALL patients were treated with an AML protocol, but none of the patients with *FLT3*wt ETP-ALL (Supplementary Table S3). The overall survival rate was similar between *FLT3*mut ETP-ALL and *FLT3*wt ETP-ALL patients (3-year survival: 58% versus 61%, P = 0.86; Figure S3 in Supplementary Figures).

### Sensitivity of T-ALL cell lines transfected with *FLT3* expression constructs to TKI

**Figure 2:**
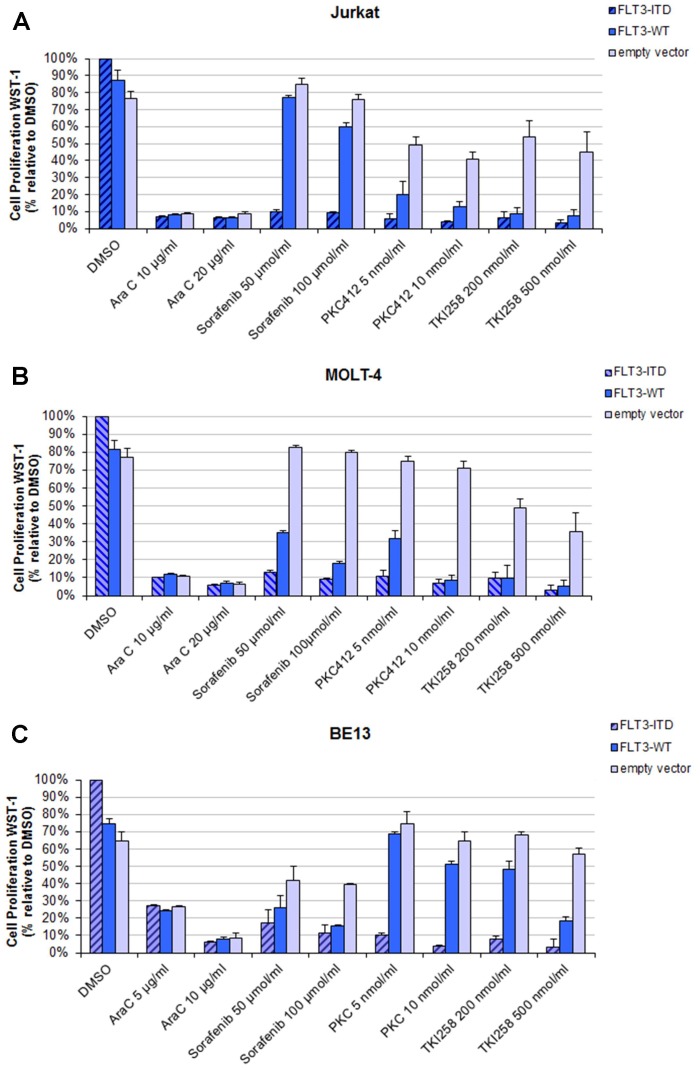
Effects of tyrosine kinase inhibitors on proliferation in T-ALL cell lines transfected with FLT3 expression constructs (A–C). Fourty-eight hours (hrs) after transfection, cells were seeded and cultured for additionally 48 hrs with tyrosine kinase inhibitors (PKC412, TKI258, and Sorafenib) and chemotherapy (AraC). Cell proliferation was measured using the WST-1 proliferation reagent. The mean optical density (OD) values corresponding to non-treated FLT3-ITD transfected cells were taken as 100%. The results were expressed in percentages of the OD of treated versus untreated control cells. Two experiments were performed in duplicates. For each drug two different doses were used. All results were expressed as means ±S.D. **A**: Jurkat cells. **B**: MOLT4 cells. **C**: BE13 cells.

In order to assess the sensitivity of TKI in a model of T-ALL with FLT3-ITD mutations, we transfected the T-ALL cell lines Jurkat, BE13 and MOLT-4 with FLT3-ITD or FLT3-wt constructs and an empty vector as control. Transfection of *FLT3* expression constructed did not alter the surface expression of myeloid (CD13, CD33) or stem cell (CD34, CD117) markers (data not shown). Cell lines transfected with FLT3-wt and FLT3-ITD constructs revealed a growth advantage compared to the empty vector transfected cells DMSO (first columns in Figure 2A–C). Treatment with TKI resulted in a selective and significant inhibition of the proliferation of FLT3-wt or FLT3-ITD transfected cells: Jurkat cells transfected with FLT3-ITD or FLT3-wt constructs showed a significant decrease in proliferation compared to empty vector transfected cells when treated with TKIs (including Sorafenib, PKC412, TKI258; Figure 2A). FLT3-ITD and FLT3-wt transfected cells were almost equally sensitive to PKC412 and TKI258, whereas empty vector transfected cells were relative insensitive. Similar results were observed for FLT3-ITD transfected MOLT-4 (Figure 2B) and BE13 cells (Figure 2C). FLT3-wt transfected cells behave different for MOLT-4 and BE13; MOLT-4 cells were more sensitive to sorafenib and BE13 cells were more resistant to PKC412 and TKI258. No differences in proliferation were observed with respect to the *FLT3* status for AraC treated cells (Figure 2A–C).

**Figure 3:**
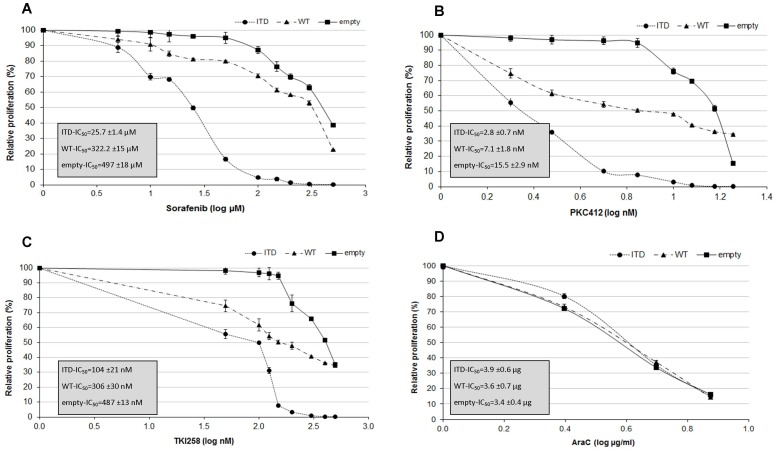
Growth inhibition of Jurkat cells transfected with FLT3 expression constructs (FLT3-ITD, FLT3-wt, and empty vector) and treated with Sorafenib, PKC412, TKI258 and AraC. IC_50_ was determined by WST-1 assay with different concentrations.

We further examined the TKI mediated apoptosis in Jurkat cells transfected with FLT3 expressing constructs. All TKIs induced enhanced apoptosis in cells transfected with FLT3 expressing constructs compared to empty vector controls (Figure S4 in Supplementary Figures). Cells treated with Sorafenib revealed a 3-fold and 2-fold increase in apoptosis in FLT3-ITD and FLT3-wt transfected cells, respectively. Similar results were observed for PKC412 and TKI258 treated cells, whereas no significant changes in apoptosis were observed for AraC (Figure S4 in Supplementary Figures). Finally, we defined the concentration (IC50) of Sorafenib, PKC412, TKI258 and AraC that induced 50% growth inhibition of Jurkat cells (Figure 3A–D). The IC50 for Sorafenib was 25.7 µM in FLT3-ITD transfected cells, compared to to 305.5 µM and 486.5 µM in FLT3-wt, and empty vector transfected cells, respectively (Figure 3A). The IC50 for PKC412 was 2.8 nM in FLT3-ITD transfected cells, compared to 7.1 nM and 15.5 nM in FLT3-wt and empty vector transfected cells, respectively (Figure 3B). Similar growth inhibitory effects were observed for TKI258 (Figure 3C). No differences in the IC50 between the different transfected cells were seen for AraC (Figure 3D).

## Discussion

In the past decades the molecular characterization of T-ALL broadly expanded and unraveled key events that drive malignant transformation. These genetic alterations may in future lead to the development and the implementation of targeted therapy. Coustan Smith et al. first identified ETP-ALL as a high risk subgroup of pediatric T-ALL characterized by a specific immature immunophenotype and a distinct gene expression profile [4]. Most recently, the genetic heterogeneity of pediatric ETP-ALL was further assessed by whole genome sequencing and next generation sequencing [6]. While various novel somatic mutations were identified, no single alteration could be detected pointing to the heterogeneous genetic background of ETP-ALL, despite the apparently common clinical and immunophenotype features. However, features shared with myeloid leukemias were present as well as mutations in genes of cytokine receptor and RAS signaling and genes involved in histone-modification were frequently observed [6]. In this work, we now focused on genes with already established prognostic and pathogenic value in AML and/or T-ALL.

We have previously characterized ETP-ALL as a high risk subgroup of early T-ALL in adults [22]. To further delineate the molecular pathomechanisms for this distinct T-ALL subgroup with stem cell like and myeloid features, we examined molecular alterations and clinical outcome in a large cohort of adult ETP-ALL patients (n = 68).

ETP-ALL is defined by a specific immunophenotype as described [4]. Recently, an additional score based on the immunophenotype was suggested to define ETP-ALL [39]. In our ETP-ALL cohort, 96% (65/68) of patients had a score greater than 6, which we used as the defining cut-off. In addition to this distinct phenotype, expression analyses of candidate genes revealed significant higher expression of stem cell associated genes and genes with adverse prognostic significance in ETP-ALL versus non-ETP T-ALL. High expression of *BAALC* and *IGFBP7*, associated with an immature high risk leukemic phenotype in adult T-ALL and AML [26], [40], underscores the immature nature of ETP-ALL. Similarly, *IGFBP7*, like *MEF2C*, were also found to be significantly upregulated in pediatric ETP-ALL [4]. In addition, *MN1* identified to be associated with ETP-ALL [32], and *WT1*, a gene known to be of unfavorable prognosis in AML as well as in T-ALL in the presence of a mutation [25], [41], were also overexpressed in the cohort of ETP-ALL.

We further observed distinct differences in the mutational profile: compared to non-ETP T-ALL, ETP-ALL patients showed less frequent *NOTCH1* mutations (15%). On the other hand, a high rate of *FLT3* mutations was observed in ETP-ALL (35%), contrasting the mutational profile of non-ETP T-ALL. *FLT3* mutations are one of the most frequent genetic alterations in AML [18], whereas *FLT3* is only infrequently mutated in leukemic lymphoblasts [42]. This underscores to some extent the association of ETP-ALL with early myeloid differentiation. In pediatric patients, a similar low rate of *NOTCH1* mutations (16%) was seen in ETP-ALL. *FLT3* mutations, although in a lower frequency (14%), occurred exclusively in ETP-ALL [6]. In addition, we analyzed the TCR rearrangement status in these ETP-ALL patients. In normal human T-cell development, TCR rearrangements are rare in prothymocytes, but are commonly found at the prethymocyte stage [43], [44]. The frequent absence of TCR rearrangement in our cohort confirms the immaturity of ETP-ALL. Together, these data further indicate that ETP-ALL represents a distinct leukemic subtype.

Interestingly, within the ETP-ALL subgroup *FLT3*mut ETP-ALL define a new molecular stem cell entity as these cases show a specific immunophenotype and molecular characteristics compared to *FLT3*wt ETP-ALL. We observed a high expression of CD2, the myeloid antigen CD13, and CD117 in the *FLT3*mut ETP-ALL. CD117, encoded by the *c-KIT* protooncogene, is highly expressed at the early stages of hematopoietic development [45], and in acute leukemia the highest frequency of CD117 expression is found in AML [46]–[48]. Expression of CD117 has been associated with *FLT3* mutations in rare cases of T-ALL [15], [16]. Here, in this yet largest cohort of *FLT3*mut T-ALL cases, only four *FLT3*mut patients lacked CD117 expression. Recently, combinations of surface markers were suggested as surrogate marker for *FLT3* mutations in T-ALL [17], [36]. However, while these combinations yield a high sensitivity, none could detect all of the ETP-ALL cases with *FLT3*mut. In our study, a combination of CD2+/CD5-/CD13+/CD33- resulted in the highest sensitivity for the presence of *FLT3* mutations in ETP-ALL with a high specificity. For the routinely performed diagnostic flow cytometry, these combinations may help to identify ETP-ALL patients that should be tested for *FLT3* mutations.

We further observed that *FLT3*mut ETP-ALL predominantly lacked clonal TCR-rearrangements pointing to a leukemic transformation before the prothymocyte stage of T-cell development. The absence of TCR rearrangements had already been linked to early treatment failure in children with T-ALL [49], providing an indirect support for the poor prognosis of ETP-ALL. The early developmental arrest of *FLT3*mut ETP-ALL is also emphasized by the low *GATA3* expression. In normal T-cell development, GATA3 plays a definite role in the early T-lineage specification as it is required for the transformation of the ETP/DN1 to the DN2a stage [34]. Thus the leukemic transformation in *FLT3*mut ETP-ALL lacking *GATA3* expression might occur at a stem cell pluripotent prothymic stage before *GATA3* expression is induced. These data in combination with the absence of activating *NOTCH1* mutations reflect an even more immature nature of the *FLT3mut* ETP-ALL within the ETP-ALL subgroup.

ETP-ALL as a subgroup of early T-ALL reflects a high risk entity with an overall survival of approximately 50% in adults [22]. Based on the findings of the GMALL study group [50], an alloSCT should be planned in first complete remission for early T-ALL patients. Even though the selection for patients undergoing alloSCT is biased due to various confounding parameters, ETP-ALL patients receiving an alloSCT showed a remarkable favorable outcome in our cohort, whereas the outcome for ETP-ALL patients receiving chemotherapy was relatively poor. The poor response to lymphoid cell-directed ALL chemotherapy only, as already reported for pediatric ETP-ALL [4], might be due to the immature nature and myeloid characteristic of the ETP-ALL. Thus, to further improve outcome for these high risk patients, in addition to alloSCT the implementation of targeted therapies should be considered. Due to the high frequency of *FLT3* mutations in ETP-ALL, TKIs already studied in *FLT3* mutated AML [51], [52] would be an attractive treatment option. We assessed the sensitivity of T-ALL cell lines transfected with FLT3-ITD and FLT3-wt expression constructs and observed that *FLT3* transfected T-ALL cells, despite of their enhanced proliferation, were particular sensitive to TKIs similar to results in AML [31]. Although the transfection of *FLT3* expression constructs in T-ALL cell lines remains an *in vitro* system, the distinct sensitivity to TKIs together with the positive experience in AML support the rational for the clinical use of TKIs in *FLT3*mut ETP-ALL. In this work, TKI side effects and the impact of TKI on the *FLT3* D835Y mutation were not evaluated. However, in analogy to AML it would be expected that the tested TKI are also able to target TKD mutations. Regarding side effects in the clinical use of TKI, the experience in AML have shown that chemotherapy backbone in combination TKIs have to be carefully chosen.

Herein, we describe that ETP-ALL patients represent a distinct molecular subgroup of adult T-ALL patients with a low frequency of *NOTCH1* mutations and a high rate of *FLT3* mutations. Moreover, we characterize *FLT3*mut ETP-ALL as a new subgroup of ETP-ALL with unique immunophenotypical and molecular features pointing to a stem cell leukemia. To further improve outcome of this high risk leukemia, targeted therapies with TKIs as well as the allocation to alloSCT should further explored.

## Supporting Information

Figure S1 **Expression of surface antigens comparing** ***FLT3*** **mut ETP-ALL patients and** ***FLT3*** **wt ETP-ALL patients.** Median and quartiles of the percentage of positive cells in the flow cytometry are pictured. Abbreviations: * statistically significant; ns, not significant.
(DOC)

Figure S2 ***FLT3*** **mRNA expression in 68 adult ETP-ALL samples measured by quantitative RT-PCR.** The *FLT3* expression was significantly higher in *FLT3*mut ETP-ALL (n = 21) compared to *FLT3*wt ETP-ALL (n = 37) (p<.01).
(DOC)

Figure S3 **Clinical outcome of** ***FLT3*** **mut ETP-ALL versus** ***FLT3*** **wt ETP-ALL patients.** The plot shown is the Kaplan Meier analysis of overall survival. P-value was calculated by the Log-Rank test.
(DOC)

Figure S4 **Effects of tyrosine kinase inhibitors on apoptosis in Jurkat cells transfected with FLT3 expression constructs.** Fourty-eight hrs after transfection the cells were cultured with tyrosine kinase inhibitors (**A**: Sorafenib, **B**: PKC412, and **C**: TKI258) or **D**: AraC. Apoptosis assay was performed by Annexin V/7AAD labeling of the cells. The results are expressed in percentage of apoptotic cells. Experiments were performed in duplicates. All results were expressed as means ±S.D.
(DOC)

Table S1 **Immunphenotype used for the classification of the 68 ETP-ALL patients.**
(DOCX)

Table S2 **Clinical characteristics of ETP-ALL patients.**
(DOCX)

Table S3 **Clinical characteristics of** ***FLT3*** **mut ETP-ALL versus** ***FLT3*** **wt ETP-ALL patients.**
(DOCX)
