# Supplementary material for: FLT3 Mutations in Early T-Cell Precursor ALL Characterize a Stem Cell Like Leukemia and Imply the Clinical Use of Tyrosine Kinase Inhibitors
Source: PLoS One. 2013 Jan 24;8(1):e53190. doi: 10.1371/journal.pone.0053190 (PMC3554732; doi:10.1371/journal.pone.0053190)
Supplement: Figure S1 — Expression of surface antigens comparing FLT3 mut ETP-ALL patients and FLT3 wt ETP-ALL patients. Median and quartiles of the percentage of positive cells in the flow cytometry are pictured. Abbreviations: * statistically significant; ns, not significant. (DOC) [file pone.0053190.s001.doc]

**Supplementary Figure S1.** Expression of surface antigens comparing *FLT3*mut ETP-ALL patients and *FLT3*wt ETP-ALL patients. Median and quartiles of the percentage of positive cells in the flow cytometry are pictured. Abbreviations: * statistically significant; ns, not significant.

* * * *  ns * ns
