# Supplementary material for: FLT3 Mutations in Early T-Cell Precursor ALL Characterize a Stem Cell Like Leukemia and Imply the Clinical Use of Tyrosine Kinase Inhibitors
Source: PLoS One. 2013 Jan 24;8(1):e53190. doi: 10.1371/journal.pone.0053190 (PMC3554732; doi:10.1371/journal.pone.0053190)
Supplement: Figure S2 — FLT3 mRNA expression in 68 adult ETP-ALL samples measured by quantitative RT-PCR. The FLT3 expression was significantly higher in FLT3mut ETP-ALL (n = 21) compared to FLT3wt ETP-ALL (n = 37) (p<.01). (DOC) [file pone.0053190.s002.doc]

**Figure S2**. *FLT3* mRNA expression in 68 adult ETP-ALL samples measured by quantitative RT-PCR. The *FLT3* expression was significantly higher in *FLT3*mut ETP-ALL (n=21) compared to *FLT3*wt ETP-ALL (n=37) (p<.01).

Expression (2^µ(ΔCT)^)
