# Supplementary material for: FLT3 Mutations in Early T-Cell Precursor ALL Characterize a Stem Cell Like Leukemia and Imply the Clinical Use of Tyrosine Kinase Inhibitors
Source: PLoS One. 2013 Jan 24;8(1):e53190. doi: 10.1371/journal.pone.0053190 (PMC3554732; doi:10.1371/journal.pone.0053190)

**Figure S3.** Clinical outcome of *FLT3*mut ETP-ALL versus *FLT3*wt ETP-ALL patients. The plot shown is the Kaplan Meier analysis of overall survival. P-value was calculated by the Log-Rank test.


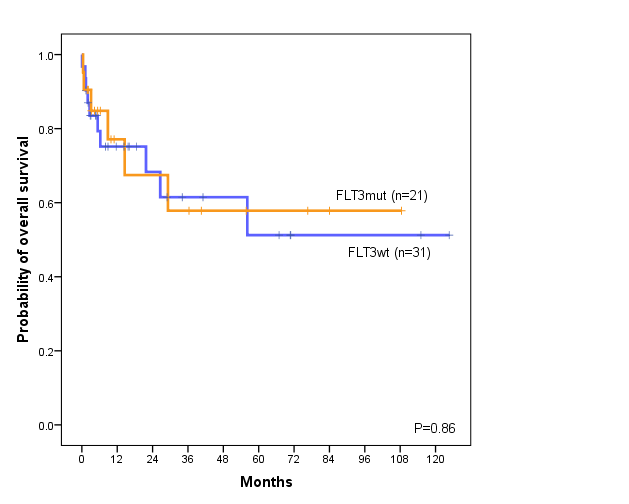

Supplement: Figure S3 — Clinical outcome of FLT3 mut ETP-ALL versus FLT3 wt ETP-ALL patients. The plot shown is the Kaplan Meier analysis of overall survival. P-value was calculated by the Log-Rank test. (DOC) [file pone.0053190.s003.doc]
