# Supplementary material for: FLT3 Mutations in Early T-Cell Precursor ALL Characterize a Stem Cell Like Leukemia and Imply the Clinical Use of Tyrosine Kinase Inhibitors
Source: PLoS One. 2013 Jan 24;8(1):e53190. doi: 10.1371/journal.pone.0053190 (PMC3554732; doi:10.1371/journal.pone.0053190)
Supplement: Figure S4 — Effects of tyrosine kinase inhibitors on apoptosis in Jurkat cells transfected with FLT3 expression constructs. Fourty-eight hrs after transfection the cells were cultured with tyrosine kinase inhibitors (A: Sorafenib, B: PKC412, and C: TKI258) or D: AraC. Apoptosis assay was performed by Annexin V/7AAD labeling of the cells. The results are expressed in percentage of apoptotic cells. Experiments were performed in duplicates. All results were expressed as means ±S.D. (DOC) [file pone.0053190.s004.doc]

**Figure S4**. Effects of tyrosine kinase inhibitors on apoptosis in Jurkat cells transfected with FLT3 expression constructs. Fourty-eight hrs after transfection the cells were cultured with tyrosine kinase inhibitors (**A:** Sorafenib, **B:** PKC412, and **C:** TKI258) or **D:** AraC. Apoptosis assay was performed by Annexin V/7AAD labeling of the cells. The results are expressed in percentage of apoptotic cells. Experiments were performed in duplicates. All results were expressed as means ±S.D.
