# Supplementary material for: FLT3 Mutations in Early T-Cell Precursor ALL Characterize a Stem Cell Like Leukemia and Imply the Clinical Use of Tyrosine Kinase Inhibitors
Source: PLoS One. 2013 Jan 24;8(1):e53190. doi: 10.1371/journal.pone.0053190 (PMC3554732; doi:10.1371/journal.pone.0053190)
Supplement: Table S1 — Immunphenotype used for the classification of the 68 ETP-ALL patients. (DOCX) [file pone.0053190.s005.docx]

**Supplementary Table S1.** Immunphenotype used for the classification of the 68 ETP-ALL patients.

|  | **Previous study** | **CD7** | **CD5** | **CD1** | **CD8** | **CD33** | **CD65s** | **CD13** | **CD15** | **CD34** | **HLADR** | **TDT** | **CD117** |
| --- | --- | --- | --- | --- | --- | --- | --- | --- | --- | --- | --- | --- | --- |
| 1 |  | 90 | 11 | 0 | 2 | 48 | 0 | 8 | 0 | 56 | 17 | 1 | 54 |
| 2 |  | 53 | 10 | 0 | 4 | 5 | 1 | 81 | 0 | 44 | 62 | 24 | 67 |
| 3 |  | 87 | 39 | 0 | 2 | 52 | 0 | 1 | -1 | 89 | 5 | 15 | 14 |
| 4 |  | 92 | 47 | 0 | 1 | 10 | 0 | 0 | -1 | 28 | 2 | 78 | 0 |
| 5 |  | 95 | 1 | 0 | 0 | 79 | -1 | 71 | 0 | 84 | 2 | 87 | 0 |
| 6 | yes | 89 | 34 | 0 | 1 | 28 | 1 | 1 | -1 | 0 | 3 | 32 | 31 |
| 7 |  | 86 | 22 | 1 | 3 | 1 | 0 | 1 | -1 | 60 | 1 | 46 | 0 |
| 8 |  | 98 | 15 | 1 | 0 | 61 | 0 | 1 | -1 | 98 | 2 | 0 | 51 |
| 9 |  | 90 | 4 | 1 | 3 | 6 | 3 | 47 | -1 | 36 | 97 | 47 | 32 |
| 10 |  | 90 | 17 | 0 | 1 | 74 | 3 | 3 | 0 | 33 | 3 | 6 | 1 |
| 11 |  | 99 | 9 | 0 | 0 | 25 | 0 | 4 | -1 | 56 | 94 | 90 | 0 |
| 12 |  | 82 | 5 | 1 | 2 | 7 | 4 | 5 | -1 | 4 | 35 | 28 | 1 |
| 13 |  | 93 | 4 | 2 | 1 | 12 | 6 | 60 | 69 | 91 | 60 | 80 | 50 |
| 14 | yes | 73 | 7 | 1 | 0 | 10 | 14 | 75 | 21 | 81 | 95 | 48 | 47 |
| 15 |  | 98 | 67 | 4 | 3 | 9 | 3 | 71 | -1 | 94 | 5 | 81 | 6 |
| 16 | yes | 97 | 73 | 0 | 0 | 5 | 0 | 0 | -1 | 69 | 90 | 90 | 0 |
| 17 |  | 97 | 66 | 0 | 3 | 20 | 0 | 1 | -1 | 1 | 45 | 10 | 0 |
| 18 |  | 95 | 66 | 0 | 2 | 51 | -1 | 95 | -1 | 75 | 21 | 69 | 25 |
| 19 |  | 52 | 3 | 0 | 0 | 2 | -1 | 63 | -1 | 33 | 69 | 45 | 26 |
| 20 |  | 77 | 59 | 0 | 0 | 46 | 0 | 62 | -1 | 0 | 1 | 63 | 42 |
| 21 |  | 93 | 7 | 0 | 1 | 2 | 0 | 7 | 0 | 15 | 87 | 95 | 24 |
| 22 | yes | 95 | 68 | 1 | 2 | 27 | 0 | 0 | -1 | 96 | 1 | 90 | 0 |
| 23 |  | 92 | 42 | 0 | 0 | 7 | -1 | 0 | -1 | 26 | 77 | 36 | 1 |
| 24 |  | 97 | 13 | 0 | 3 | 1 | 1 | 1 | 1 | 55 | 58 | 54 | 17 |
| 25 | yes | 98 | 20 | 1 | 3 | 96 | 3 | 0 | 3 | 84 | 60 | 95 | 96 |
| 26 | yes | 98 | 53 | 0 | 2 | 39 | -1 | 45 | -1 | 86 | 5 | 52 | 0 |
| 27 |  | 49 | 1 | 0 | 0 | 3 | 20 | 66 | 3 | 55 | 96 | 69 | 86 |
| 28 |  | 79 | 10 | 0 | 2 | 7 | 7 | 87 | 4 | 93 | 38 | 55 | 87 |
| 29 |  | 66 | 11 | 0 | 3 | 6 | -1 | 69 | -1 | 57 | 77 | 46 | 63 |
| 30 |  | 96 | 41 | 1 | 0 | 78 | -1 | 16 | -1 | 90 | 4 | 84 | 8 |
| 31 |  | 90 | 59 | 1 | 1 | 97 | 7 | 29 | 4 | 85 | 72 | 2 | 1 |
| 32 |  | 93 | 2 | 1 | 2 | 75 | 0 | 64 | 0 | 20 | 62 | 46 | 8 |
| 33 |  | 98 | 7 | 0 | 1 | 4 | -1 | 21 | -1 | 7 | 44 | 73 | 2 |
| 34 |  | 83 | 45 | 0 | 1 | 94 | 0 | 36 | 0 | 52 | 37 | 60 | 0 |
| 35 |  | 97 | 49 | 0 | 2 | 2 | -1 | 3 | 1 | 1 | 99 | 85 | 5 |
| 36 | yes | 78 | 4 | 0 | 2 | 7 | 17 | 63 | 35 | 59 | 13 | 35 | 43 |
| 37 |  | 71 | 2 | 1 | 2 | 8 | 22 | 47 | 3 | 82 | 75 | 48 | 42 |
| 38 |  | 90 | 9 | 0 | 2 | 6 | 12 | 91 | 4 | 6 | 58 | 33 | 18 |
| 39 |  | 99 | 49 | 0 | 3 | 44 | -1 | 0 | -1 | 82 | 82 | 79 | 3 |
| 40 |  | 82 | 3 | 1 | 1 | 1 | 13 | 31 | 6 | 52 | 69 | 52 | 85 |
| 41 |  | 96 | 4 | 1 | 1 | 5 | -1 | 77 | -1 | 74 | 94 | 52 | 2 |
| 42 |  | 74 | 3 | 0 | 2 | 95 | 3 | 53 | 1 | 92 | 64 | 95 | 62 |
| 43 |  | 95 | 5 | 1 | 2 | 0 | 1 | 71 | 1 | 90 | 52 | 32 | 89 |
| 44 |  | 98 | 66 | 0 | 3 | 80 | 0 | 0 | 0 | 68 | 88 | 86 | 1 |
| 45 |  | 97 | 6 | 0 | 1 | 3 | -1 | 0 | -1 | 61 | 7 | 30 | 0 |
| 46 |  | 94 | 4 | 0 | 1 | 4 | 17 | 72 | 21 | 19 | 52 | 30 | 29 |
| 47 |  | 64 | 3 | 1 | 2 | 1 | 3 | 70 | 3 | 43 | 80 | 26 | 85 |
| 48 |  | 99 | 22 | 1 | 4 | 3 | -1 | 64 | -1 | 95 | 1 | 44 | 0 |
| 49 |  | 99 | 2 | 0 | 1 | 82 | 1 | 0 | 2 | 85 | 1 | 64 | 21 |
| 50 |  | 98 | 16 | 1 | 4 | 4 | -1 | 33 | -1 | 20 | 42 | 55 | 2 |
| 51 |  | 98 | 28 | 0 | 0 | 95 | -1 | 0 | -1 | 22 | 41 | 34 | 0 |
| 52 |  | 98 | 2 | 4 | 1 | 2 | 45 | 85 | 72 | 87 | 97 | 42 | 59 |
| 53 |  | 89 | 2 | 1 | 1 | 3 | 21 | 27 | 10 | 14 | 79 | 39 | 44 |
| 54 |  | 95 | 7 | 0 | 1 | 1 | 9 | 95 | 15 | 77 | 98 | 85 | 62 |
| 55 |  | 93 | 9 | 2 | 3 | 5 | -1 | 5 | -1 | 3 | 7 | 71 | 42 |
| 56 |  | 97 | 42 | 1 | 2 | 9 | -1 | 68 | -1 | 0 | 19 | 65 | 3 |
| 58 |  | 91 | 12 | 4 | 4 | 7 | -1 | 52 | -1 | 55 | 10 | 69 | 1 |
| 59 |  | 72 | 6 | 0 | 1 | 0 | 12 | 95 | 16 | 91 | 98 | 24 | 0 |
| 60 |  | 74 | 13 | 0 | 2 | 15 | 20 | 87 | 20 | 96 | 91 | 22 | 63 |
| 62 | yes | 88 | 3 | 0 | 1 | 16 | 3 | 56 | 6 | 57 | 68 | 49 | 74 |
| 64 | yes | 95 | 15 | 0 | 3 | 39 | -1 | 4 | -1 | 76 | 40 | 45 | 0 |
| 65 | yes | 96 | 29 | 0 | 3 | 77 | -1 | 3 | -1 | 88 | 42 | 44 | 4 |
| 66 | yes | 90 | 4 | 0 | 2 | 1 | 4 | 66 | 20 | 59 | 93 | 85 | 80 |
| 67 | yes | 97 | 71 | 0 | 1 | 2 | -1 | 1 | -1 | 93 | 3 | 5 | 0 |
| 68 | yes | 93 | 1 | 0 | 1 | 0 | 1 | 97 | 1 | 80 | 20 | 53 | 71 |
| 69 | yes | 98 | 24 | 4 | 2 | 3 | -1 | 48 | -1 | 1 | 3 | 43 | 4 |
| 70 | yes | 95 | 4 | 0 | 3 | 1 | -1 | 31 | -1 | 2 | 49 | 49 | 57 |
| 71 | yes | 99 | 4 | 0 | 1 | 95 | 1 | 16 | 0 | 97 | 1 | 95 | 44 |

Abbreviations: “-1”, not determined.
