# Supplementary material for: FLT3 Mutations in Early T-Cell Precursor ALL Characterize a Stem Cell Like Leukemia and Imply the Clinical Use of Tyrosine Kinase Inhibitors
Source: PLoS One. 2013 Jan 24;8(1):e53190. doi: 10.1371/journal.pone.0053190 (PMC3554732; doi:10.1371/journal.pone.0053190)
Supplement: Table S2 — Clinical characteristics of ETP-ALL patients. (DOCX) [file pone.0053190.s006.docx]

**Supplementary Table S2.** Clinical characteristics of ETP-ALL patients.

|  |  | **ETP-ALL** |
| --- | --- | --- |
| Number of patients | n= | 68 |
| Sex | male | 55 |
|  | female | 13 |
| Age (years) | median | 38 |
|  | range | 17-74 |
| Clinical information available | n= | 52 |
| Allogeneic SCT | yes | 20 |
|  | no | 19 |
|  | unknown | 13 |
| Induction therapy | ALL-like | 45 |
|  | AML-like | 3 |
|  | unknown | 4 |
| Outcome of induction | CR | 26 (50%) |
|  | PR | 5 (10%) |
|  | stopped | 2 (4%) |
|  | refractory | 9 (17%) |
|  | death | 3 (6%) |
|  | unknown | 7 (13%) |

Abbreviations: CR, complete remission; PR, partial remission.
