# Supplementary material for: FLT3 Mutations in Early T-Cell Precursor ALL Characterize a Stem Cell Like Leukemia and Imply the Clinical Use of Tyrosine Kinase Inhibitors
Source: PLoS One. 2013 Jan 24;8(1):e53190. doi: 10.1371/journal.pone.0053190 (PMC3554732; doi:10.1371/journal.pone.0053190)
Supplement: Table S3 — Clinical characteristics of FLT3 mut ETP-ALL versus FLT3 wt ETP-ALL patients. (DOCX) [file pone.0053190.s007.docx]

**Supplementary Table S3.** Clinical characteristics of *FLT3*mut ETP-ALL versus *FLT3*wt ETP-ALL patients

|  |  | | | **FLT3mut** | **FLT3wt** |
| --- | --- | --- | --- | --- | --- |
| Number of patients | | | | 24 | 44 |
| Sex | | male | | 21 | 34 |
|  | | female | | 3 | 10 |
| Age (years) | | median | | 41 | 37 |
|  | | range | | 17-73 | 18-74 |
| Information available | | | | 21 | 31 |
| AlloSCT performed | | | yes | 6 | 14 |
|  | | | no | 10 | 9 |
|  | | | unknown | 5 | 8 |
| Induction therapy | | | ALL protocol | 18 | 27 |
|  |  |  | AML protocol | 3 | 0 |
|  | | | unknown | 0 | 4 |
| Outcome of induction | | | CR | 13 | 13 |
|  |  |  | PR | 3 | 2 |
|  | | | stopped | 2 | 0 |
|  | | | refractory | 1 | 8 |
|  | | | death | 2 | 1 |
|  | | | unknown | 0 | 7 |

Abbreviations: CR, complete remission; PR, partial remission; alloSCT, allogeneic hematopoietic stem cell transplantation.
